# Reprogramming and differentiation-dependent transcriptional alteration of DNA damage response and apoptosis genes in human induced pluripotent stem cells

**DOI:** 10.1093/jrr/rrz057

**Published:** 2019-10-28

**Authors:** Mikio Shimada, Kaima Tsukada, Nozomi Kagawa, Yoshihisa Matsumoto

**Affiliations:** Laboratory for Advanced Nuclear Energy, Institute of Innovative Research, Tokyo Institute of Technology, 2-12-1, Oookayaka, Meguro-ku, 152-8550, Tokyo, Japan

**Keywords:** iPS cells, neural progenitor cells, DNA damage response, apoptosis

## Abstract

Pluripotent stem cells (PSCs), such as embryonic stem cells (ESCs) and induced pluripotent stem cells (iPSCs), have a dual capability to self-renew and differentiate into all cell types necessary to develop an entire organism. Differentiation is associated with dynamic epigenetic alteration and transcriptional change, while self-renewal depends on maintaining the genome DNA accurately. Genome stability of PSCs is strictly regulated to maintain pluripotency. However, the DNA damage response (DDR) mechanism in PSCs is still unclear. There is accumulating evidence that genome stability and pluripotency are regulated by a transcriptional change in undifferentiated and differentiated states. iPSCs are ideal for analyzing transcriptional regulation during reprogramming and differentiation.

This study aimed to elucidate the transcriptional alteration surrounding genome stability maintenance, including DNA repair, cell cycle checkpoints and apoptosis in fibroblasts, iPSCs and neural progenitor cells (NPCs) derived from iPSCs as differentiated cells. After ionizing radiation exposure, foci for the DNA double-stranded break marker γ-H2AX increased, peaking at 0.5 h in all cells (>90%), decreasing after 4 h in fibroblasts (32.3%) and NPCs (22.3%), but still remaining at 52.5% (NB1RGB C2 clone) and 54.7% (201B7 cells) in iPSCs. Terminal deoxynucleotidyl transferase dUTP nick end labeling (TUNEL)-positive cells were detected, indicating that iPSCs’ apoptosis increases. In addition, RNA sequencing (RNA-Seq) analysis showed high expression of apoptosis genes (*TP53*, *CASP3* and *BID*) in iPSCs. Results suggested that increased apoptosis activity maintains accurate, undifferentiated genome DNA in the cell population.

## INTRODUCTION

Pluripotent stem cells (PSCs), including embryonic stem cells (ESCs) and induced pluripotent stem cells (iPSCs), have a dual capability to self-renew and differentiate into all cell types necessary to develop an entire organism. Differentiation is associated with dynamic epigenetic alteration and transcriptional change, while self-renewal depends on accurately maintaining the genome DNA. PSCs are permanently exposed to endogenous genotoxic stress factors, such as reactive oxygen species (ROS), which damage DNA. The DNA damage response (DDR) is highly organized by several molecular mechanisms, such as DNA repair, cell cycle checkpoints and apoptosis (programed cell death), because DNA damage causes mutation and genome instability [1–5]. The transcriptional regulation of related genes is altered in somatic cells and PSCs. PSCs maintain a high rate of proliferation [6, 7]. Studies have reported that reprogramming is highly stressful for cells and causes accumulated DNA damage [5, 8, 9]. The knockdown of p53, a master regulator of DDR, increases reprogramming efficiency because DDR acts as a barrier to mutation in genome DNA [10, 11]. Ionizing radiation (IR) also induces DNA damage, such as base-excision damage, single-strand breaks and double-strand breaks (DSBs). DSBs, in turn, cause chromosome aberration and apoptosis. There are two main pathways to DSB repair: (i) homologous recombination (HR) repair and (ii) nonhomologous end joining (NHEJ) repair. NHEJ repair joins DNA ends directly without the need for DNA templates, while HR repair requires a sister chromatid to act as a DNA template. Although the HR repair pathway is limited to the S-G2 cell cycle phase, its activity is maintained at a high level in PSCs [2, 12, 13]. Cells derived from patients with an inherited disease (e.g., Fanconi anemia [FA], ataxia-telangiectasia-like disorder [ATLD], Nijmegen breakage syndrome [NBS] and ligase IV syndrome) involving a defect in DNA repair proteins (FA proteins, ataxia-telangiectasia-mutated [ATM], NBS1 and ligase IV, respectively) show low reprogramming efficiency [14–19], indicating that the DNA repair pathway is also involved in reprogramming. In addition, defective DNA repair mechanisms are associated with a variety of neurodegenerative conditions [20–22]. For example, ATLD is characterized by progressive loss of cerebellar Purkinje and granule neurons, while FA, NBS and ligase IV syndrome patients show microcephaly, which is associated with developmental defects in the cerebral cortex [21, 23]. *In utero* IR exposure induces DNA damage, which critically affects brain development in mice [24]. Neural progenitor cells (NPCs), in particular, are hypersensitive to such DNA damage. Therefore, DNA repair is important for neural development, although details remain unclear.

It has been reported that PSCs are hypersensitive to DNA damage and apoptosis [3, 25]. In human ESCs (hESCs), Bax (the pro-apoptotic member of the Bcl-2 family) is constitutively activated and located in the Golgi body [26]. Because of DNA damage, active Bax translocates to the mitochondria in a p53-dependent manner; this does not occur in differentiated cells [27]. In human induced pluripotent stem cells (hiPSCs), the expression levels of the anti-apoptotic factors *BCL2*, *BCLX*, *BCLW* and *BCLA1* are down-regulated [28, 29]. These data suggest a low threshold of PSC apoptosis.

In this study, to elucidate DDR transcriptional alteration between PSCs and differentiated cells, we generated iPSCs and NPCs from fibroblasts and investigated their sensitivity to DNA damage. We further analyzed transcriptional profiles of PSCs using next-generation RNA sequencing (RNA-Seq) analysis. The present results indicated a high tendency of apoptosis of PSC in response to DNA damage and its possible underlying mechanisms, that is enhanced apoptosis-related genes expression (*CASP3* and *BID*), and, on the other hand, attenuation of the expression of cell cycle checkpoint-related genes (e.g. *CDKN1A*). A differential expression pattern of DNA repair genes, such as NBS1 (*NBN*) and MRE11 (*MRE11A*), in iPSCs and NPCs was also found, which might be associated with neural disease development.

## MATERIALS AND METHODS

### Cell culture

The human skin fibroblast cell line NB1RGB was obtained from the RIKEN BioResource Center (Ibaraki, Japan). The NB1RGB was maintained in Dulbecco’s modified Eagle’s medium (DMEM; Nacalai Tesque, Japan) supplemented with 10% fetal bovine serum (FBS; Hyclone, GE Healthcare, Chicago, IL, USA) and penicillin/streptomycin (Nacalai Tesque) at 37°C under 5% CO_2_ conditions. The iPSCs derived from NB1RGB were cultured by feeder-free methods using precoating with iMatrix511 (Nippi, Japan) and maintained in NutriStem™ XF/FF (Stemgent, Beltsville, MD, USA) culture medium at 37°C under 5% CO_2_ conditions. The hiPSC cell line 201B7 was obtained from the RIKEN BioResource Center and adapted from a fed-batch culture to a feeder-free culture using iMatrix and NutriStem™ XF/FF. NPCs derived from iPSCs were maintained in a PSC neural basal medium (Gibco, Thermo Fisher Scientific, Waltham, MA, USA). To prevent apoptosis, the iPSC and NPC culture media were supplemented with the Y27632 ROCK inhibitor (WAKO Pure Chemical Industries, Tokyo, Japan) during passage; on the subsequent day, the media were replaced again but without the Y27632 ROCK inhibitor.

### Generation of iPSCs

iPSCs were derived from NB1RGB by messenger RNA (mRNA) integration-free methods using the Stemgent® StemRNA™-NM Reprogramming Kit for Reprogramming Adult and Neonatal Human Fibroblasts (Stemgent, Cambridge, MA, USA). iPSC induction was performed according to the manufacturer’s instructions. Briefly, NB1RGB was plated on iMatrx511-precoated 6-well plates and maintained in DMEM supplemented with 10% FBS and penicillin/streptomycin. On the following day, the culture medium was replaced with the NutriStem™ XF/FF. A non-modified RNA (NM-RNA) reprogramming cocktail comprising, OSKMNL NM-RNA (Oct4, Sox2, Klf4, cMyc, Nanog and Lin28 reprogramming factors); E3, K3 and B18 immune evasion factors (EKB) NM-RNA; and NM-microRNAs, were transfected with the Lipofectamine® RNAiMAX™ transfection reagent (Invitrogen, Thermo Fisher Scientific, USA). After the initial transfection, the NutriStem™ XF/FF culture medium was replaced and the NM-RNA reprogramming cocktail was transfected daily for 4 days. Five days after the initial transfection, the culture medium was replaced with the NutriStem™ XF/FF. Ten days after the initial transfection, iPSC-like colonies were transferred to an iMatrix511-precoated 12-well plates and incubated.

### Generation of NPCs

NPCs were derived from iPSCs using the PSC neural induction medium (Gibco, Thermo Fisher Scientific) and maintained with a neural basal medium (Gibco, Thermo Fisher Scientific) according to the manufacturer’s instructions with small modifications. Briefly, iPSCs were placed on iMatrix511-precoated 6-well plates and grown to 15–25% confluency, and maintained in NutriStem™ XF/FF with ROCK inhibitor. On the following day, the culture medium was replaced with the PSC neural induction medium and then replaced again every 2 days. Seven days after neural induction, cells were expanded with a neural expansion medium, including ROCK inhibitor, as passage 0 (P0). After passage 1, the cells were used in experiments as NPCs.

### Alkaline phosphatase staining

To confirm pluripotency, iPSC colonies were stained using a Leukocyte Alkaline Phosphatase kit (Sigma-Aldrich, St. Luis, MO, USA).

### Germ-layer differentiation and identification

iPSCs were differentiated into the three germ layers using a Human Pluripotent Stem Cell Functional Identification Kit (R&D systems, Minneapolis, MN, USA) according to the manufacturer’s instructions. To confirm differentiation, the cells were immunostained with Otx2, Brachury, and SOX17 antibodies (R&D systems) as ectoderm, mesoderm and endoderm germ-layer markers, respectively.

### Western blotting

Cells were lysed with a radioimmunoprecipitation assay (RIPA) buffer (50 mM Tris HCl, pH 8.0; 250 mM NaCl; 25 mM ethylenediaminetetraacetic acid [EDTA]; 0.5% Triton X-100; 0.5% sodium dodecyl sulfate [SDS]; and 0.5% sodium deoxycholate), and the protein concentration was measured using a bicinchoninic acid (BCA) assay kit (Takara Bio, Shiga, Japan). Protein (20 μg) was loaded onto SDS polyacrylamide gel electrophoresis (SDS-PAGE) plates, and electrophoresed at 30 mA/gel plate for 1 h, and transferred to a polyvinylidene fluoride (PVDF) membrane at 100 V for 1.5 h. Next, the PVDF membrane was blocked with 1% skim milk at room temperature for 1 h. The following primary antibodies were reacted with the PVDF membrane at 4°C for 4–24 h: ATM (mouse, 1:1000, Sigma Aldrich, cat# A1106), KAP1 (rabbit, 1:1000, Abcam, UK, cat# ab10484), S824P KAP1 (rabbit, 1:1000, Bethyl, USA, cat# A300-767A), p53 (mouse, 1:2000, Santa Cruz Biotechnology, USA, cat# sc-126), S15P p53 (rabbit, 1:1000, Cell Signaling Technology, USA, cat# 9284), CHK1 (mouse, 1:1000, Santa Cruz Biotechnology, USA, cat# sc-8408), p21 (mouse, 1:1000, BD Biosciences, USA, cat# 556431), NBS1 (rabbit, 1:1000, Proteintech, USA, cat# 55025–1-AP) and glyceraldehyde-3-phosphate dehydrogenase (GAPDH) (mouse, 1:2000, Chemicon, Merck Millipore, Germany, cat#MAB374). The PVDF membrane was rinsed three times with tris-buffered saline and Tween20 (TBS-T). Horseradish peroxidase (HRP)-conjugated rabbit or mouse antibodies were used as secondary antibodies. The PVDF membrane was developed by enhanced chemiluminescence (LI-COR, Biosciences, Lincoln, NE, USA) and detected by C-digit (LI-COR). Full scan data are provided in Supplementary Figs 3 and 4.

### Immunofluorescence

NB1RGB cells were cultured on a cover glass. iPSC and NPC cells were cultured on 24-well plates. After DNA damage treatment, cells were washed twice with phosphate-buffered saline (PBS) and fixed with 4% paraformaldehyde (PFA) at room temperature for 10 min. After washing twice with PBS, the cells were permeabilized with 0.5% Triton X-100 in PBS at 4°C for 5 min, blocked with 1% bovine serum albumin/PBS-Tween 20 (BSA/PBS-T) at room temperature for 30 min and reacted with the following primary antibodies at 4°C for 1 h: S139P γ-H2AX (mouse, 1:1000, Merck Millipore, cat# 05–636), 53BP1 (rabbit, 1:1000, Bethyl, cat# A300-272A). After washing three times with PBS-T, the cells were reacted with Alexa Fluor 488−/594-conjugated secondary antibodies (Thermo Fisher Scientific), then washed five times with PBS-T, mounted with a mounting medium (Dako, Agilent, Carpinteria, CA, USA) and counterstained with 4′,6-diamidino-2-phenylindole (DAPI). Images were captured using an Olympus fluorescence microscope (Olympus Corporation, Tokyo, Japan).

### TUNEL assay

Apoptosis was measured using an Apoptag Fluorescent Direct *in situ* Apoptosis Detection Kit (Merck Millipore; cat# S7160) according to the manufacturer’s instructions. After terminal deoxynucleotidyl transferase dUTP nick end labeling (TUNEL) staining, the cells were counterstained with DAPI.

### Colony formation assay

Cell survival was determined using the colony formation assay. NB1RGB cells were plated on 60-mm dishes and irradiated with the designated doses. iPSCs were plated on iMatrix 511-precoated 60-mm dishes in NutriStem™ XF/FF culture medium with the Y27632 ROCK inhibitor. On the subsequent day, the medium was replaced with fresh medium without the ROCK inhibitor and irradiated. After 10–14 days, cells were fixed with 100% ethanol and stained with crystal violet. All experiments were repeated at least three times.

### RNA sequencing

An hour after the 5 Gy IR treatment had been applied to the NB1RGB, NB1RGB C2 and NB1RGB NPCs C2, total RNA was extracted using the Fast Gene RNA premium kit (Nippon Genetics Co. Ltd., Tokyo, Japan). RNA-seq was done by Eurofins Genomics (Tokyo, Japan). For RNA-seq data analysis, FASTQ data were uploaded on the Illumina BaseSpace Sequence Hub. Quality check and quality control of the FASTQ file were performed using the FASTQ toolkit and FAST QC, respectively. Low-quality bases were trimmed from both ends and trimmed reads were aligned to the reference genome hg19 using TopHat (Bowtie2). Gene differential expression profiles were obtained using Cufflinks Assembly & DE and indicated as fragments per kilobase of exon per million reads mapped (FPKM). Heat maps were obtained using gene differential expression profiles.

### Accession number

RNA-seq data in this study have been deposited in the National Center for Biotechnology Information (NCBI) Gene Expression Omnibus with accession number GSE113125.

### IR exposure

For IR treatment, ^60^Co was used as a gamma-ray source at the Tokyo Institute of Technology (Tokyo, Japan).

### Quantification and statistical analysis

We quantified 53BP1, γ-H2AX foci-positive cells and TUNEL-positive cells. All experiments were performed at least three times. Statistical analysis was performed using Welch’s (one tailed) *t*-test and Microsoft Excel.

## RESULTS

### Generation of iPSCs and NPCs from human skin fibroblasts

**Fig. 1:**
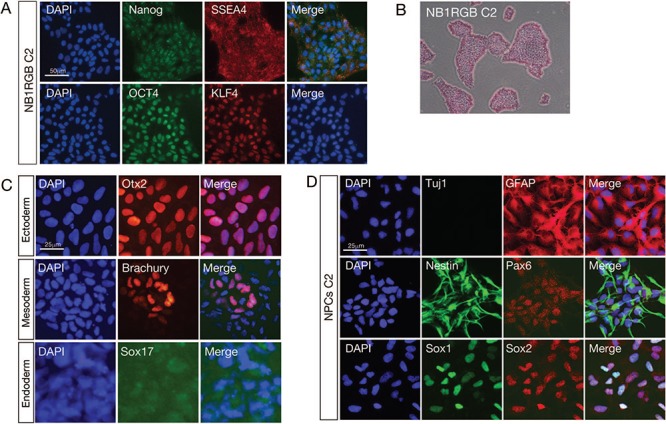
**Generation of iPSCs and NPCs from human skin fibroblasts (NB1RGB).** (**A**) To identify the PSC status of the established iPSC clone 2 (C2) from human skin fibroblasts (NB1RGB), cells were immunostained using PSC markers Nanog, SSEA4, OCT4 and KLF4. The cell nucleus was counterstained with 4′,6-diamidino-2-phenylindole (DAPI). (**B**) iPSC-like colonies were stained with alkaline phosphatase staining. (**C**) To confirm pluripotency, iPSCs were differentiated into three germ layers and stained with germ-layer markers Otx2, Brachury and Sox17 for the ectoderm, mesoderm and endoderm, respectively. (**D**) iPSCs were differentiated to neural progenitor cells (NPCs) and stained with neural stem cell markers GFAP, Nestin, Pax6, Sox1 and Sox2 and the differentiated neuron marker β-III Tubulin (Tuj1). NPCs were positive for all the NPC markers except Tuj1. Scale bar represents 25 μm or 50 μm, as indicated. iPSCs = induced pluripotent stem cells, NPCs = neural progenitor cells, PSC, pluripotent stem cell = DAPI, 4′,6-diamidino-2-phenylindole.

To investigate the PSCs’ genome maintenance mechanism and compare it to differentiated cells, we established iPSCs from human skin fibroblasts, NB1RGB, which have <20 passages and maintain a comparatively high proliferation activity. To avoid the integration of reprogramming factors into the genome DNA, the induction protocol we used was mRNA transfection. More than 100 iPSC-like colonies were obtained, ten of which were selected and maintained by feeder-free culture. Established iPSCs were named NB1RGB iPSC clones 1–10 (C1–10). To confirm the stem cell state of the iPSC clones, first, Nanog, the octamer-binding transcription factor 3/4 (OCT4), stage-specific embryonic antigen 4 (SSEA4) and Krupple-like factor 4 (KLF4) antibodies were used in immunostaining as PSC markers (Fig. 1A, Supplementary Fig. 1A). All of the iPSC clones were found to be positive for the PSC markers. Second, alkaline phosphatase staining was performed to confirm the stem cell state of the iPSC clones (Fig. 1B). To identify pluripotency, we attempted to induce the differentiation of the NB1RGB C2 clone, which maintains a high proliferation activity, into three germline layers: ectoderm, mesoderm and endoderm. Differentiated cells were confirmed by immunostaining for the antibodies Otx2 (ectoderm), Brachury (mesoderm) and Sox17 (endoderm) (Fig 1C). To address DDR’s role in neural nervous system, we generated NPCs from NB1RGB C2 and C3 clones. Established NPCs were positive for neural stem cell markers such as nestin, paired box domain (Pax6), SRY-box 1/2 (Sox1, Sox2) and glial fibrillary acidic protein (GFAP), but negative for neuron marker β-III Tubulin (Tuj1) (Fig. 1D and Supplementary Fig. 1B).

**Fig. 2:**
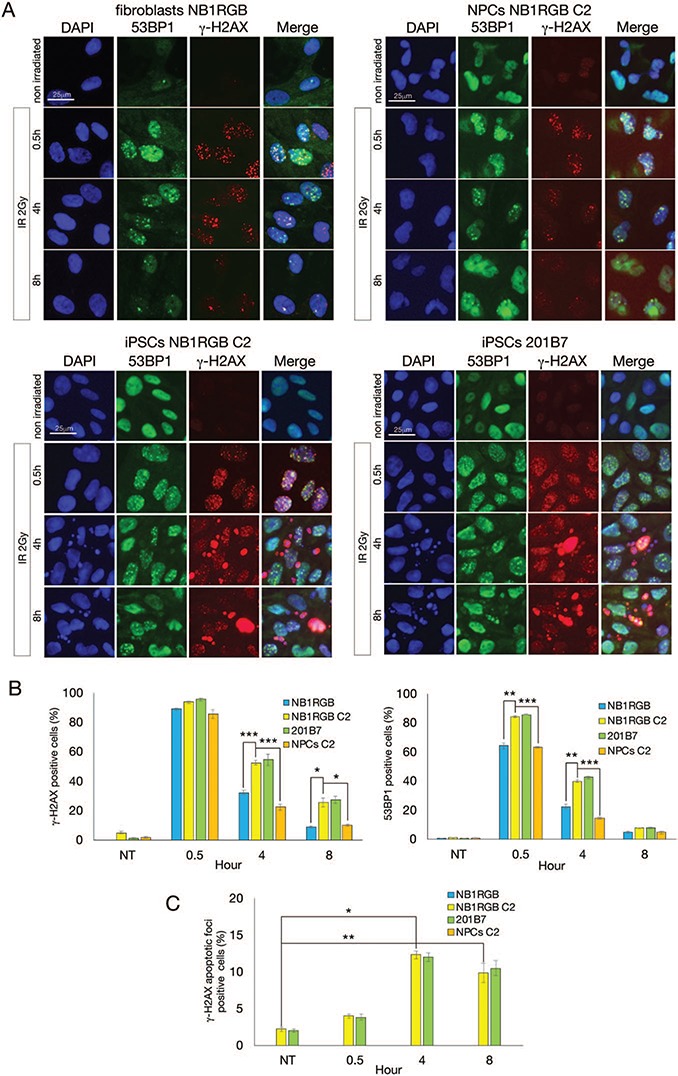
**DDR after IR in iPSCs and NPCs.** (**A**) Fibroblasts (NB1RGB), iPSCs (NB1RGB C2 and 201B7) and NPCs (C2) were irradiated at 2 Gy and fixed with 4% PFA at designated times. Cells were immunostained with 53BP1 (green) and γ-H2AX (red) antibodies. The cell nucleus was counterstained with DAPI. (**B**) Immunostained cells containing >10 53BP1- and γ-H2AX-positive foci (dot foci) were counted and graphed. (**C**) γ-H2AX apoptotic foci (pan staining) were also counted. At least 200 cells were counted and all experiments were performed three times. Scale bar represents 25 μm. Error bars represent the standard error of the mean (SEM). Welch’s (one-tailed) *t*-test was performed and statistical significance is indicated by asterisks (^*^P < 0.05; ^**^P < 0.01; ^***^P < 0.001). DDR = DNA damage response, IR = ionizing radiation, iPSCs = induced pluripotent stem cells, NPCs = neural progenitor cells, PFA = paraformaldehyde, 53BP1 = p53 binding protein 1, γ-H2AX = γ-H2A histone family member X, DAPI = 4′,6-diamidino-2-phenylindole.

**Fig. 3:**
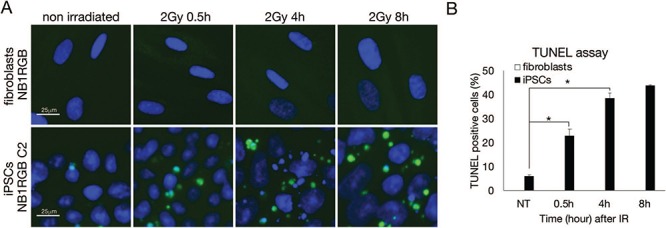
**Apoptosis detection by TUNEL assay in iPSCs.** (**A**) Fibroblasts (NB1RGB) and iPSCs (NB1RGB C2) were irradiated at 2 Gy and fixed at designated times after IR. Apoptosis was detected via the TUNEL assay. (**B**) At least 200 cells were counted and all experiments were performed three times and graphed. Scale bar represents 25 μm. Error bars represent the SEM. Welch’s (one-tailed) *t*-test was performed and statistical significance is indicated by asterisks (^*^P < 0.05). iPSCs = induced pluripotent stem cells, TUNEL = terminal deoxynucleotidyl transferase dUTP nick end labeling, SEM = standard error of the mean.

### DNA repair activity and apoptosis in iPSCs and NPCs

Previous studies have reported that DDR is highly activated in mouse embryonic stem cells (mESCs), hESCs, mouse induced pluripotent stem cells (miPSCs) and hiPSCs [1, 2, 4]. To confirm this, we used two hiPSCs, the NB1RGB C2 clone and 201B7 cells, previously reported by Takahashi *et al.* [6]. 201B7 cells were adapted for feeder-free culture. Skin fibroblasts NB1RGB and NPCs that were derived from the NB1RGB C2 clone were used as differentiated cells. After 2 Gy IR exposure, cells were fixed at a designated time and immunostained with p53 binding protein 1 (53BP1) and γ-H2AX antibodies (Fig. 2A). 53BP1 acts as a DSB repair mediator, promoting NHEJ and suppressing HR [30]. The phosphorylation of H2AX at serine 139 was catalysed by ATM and DNA-dependent protein kinase catalytic subunits (DNA-PKcs) to activate DSB repair and used as a DSB marker. Interestingly, despite an increase in 53BP1-positive foci in iPSCs at 4 h (NB1RGB C2: 40.0%, 201B7: 42.5%) after IR, most foci had disappeared at 8 h (NB1RGB C2: 7.5%, 201B7: 7.8%) after IR (Fig. 2B). Meanwhile, γ-H2AX foci peaked at 0.5 h after IR in all cells and remained at higher levels in iPSCs (NB1RGB C2: 52.5%, 201B7: 54.7%) than in fibroblasts (32.3%) and NPCs (22.3%) at 4 h after IR. Because γ-H2AX represents DNA ends and pan staining of γ-H2AX means late stage of apoptosis, pan staining of γ-H2AX is used as an apoptotic marker. We found apoptotic γ-H2AX foci even in untreated iPSCs and found an increase at 4 h (NB1RGB: 0.0%, NB1RGB C2: 12.3%, 201B7: 12.0%, NPCs: 0.0%) and 8 h (NB1RGB: 0.0%, NB1RGB C2: 9.8%, 201B7: 10.5%, NPCs: 0.0%) after IR in iPSCs but not in fibroblasts and NPCs (Fig. 2C). The apoptosis activity was confirmed by TUNEL assay (Fig. 3). TUNEL assay revealed significant apoptosis at 0.5 h (NB1RGB: 0.0%, NB1RGB C2: 23.0%), 4 h (NB1RGB: 0.0%, NB1RGB C2: 38.7%) and 8 h (NB1RGB: 0.0%, NB1RGB C2: 43.8%) after IR in iPSCs but not in fibroblasts. TUNEL assay detected the 3′-OH termini of DNA breaks and a wider range of apoptotic cells compared with γ-H2AX foci. These results are consistent with the hESC phenotype [27], suggesting that dominant apoptosis activity removes cells with damaged DNA from the iPSC population.

### RNA sequencing revealed differential expression of DDR genes in iPSCs

**Fig. 4:**
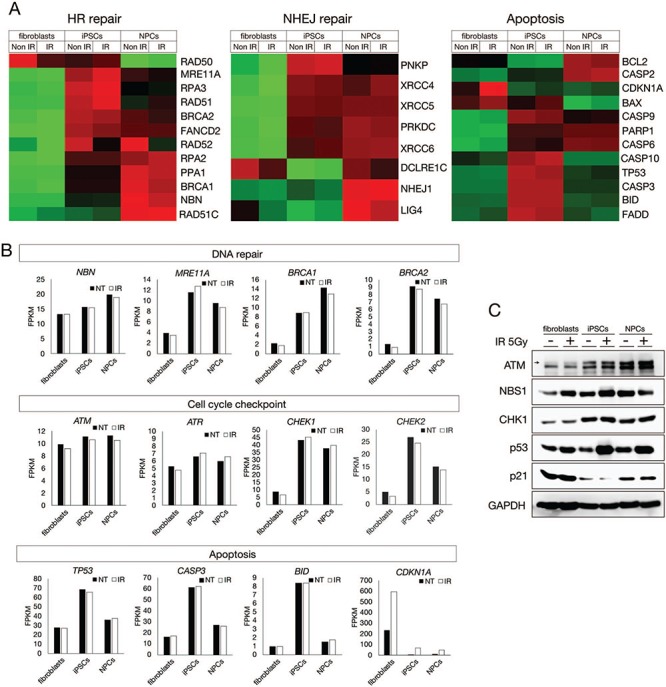
**RNA-Seq analysis revealed transcriptional alteration in fibroblasts, iPSCs and NPCs.** (**A**) Fibroblasts (NB1RGB), iPSCs (NB1RGB C2 and 201B7) and NPCs (C2) were irradiated at 5 Gy. One hour after IR, RNA samples were extracted and analyzed using the Illumina HiSeq 2500 next-generation sequencer for RNA-Seq. Raw data were quality controlled and aligned to the reference hg19. Heat maps classed as HR repair, NHEJ repair and apoptosis were obtained using R software. Red, upregulated; green, downregulated. (**B**) Differential expression patterns of each gene were represented by FPKM. Expression differences were classified according to molecular machinery components: DNA repair, cell cycle checkpoints and apoptosis. (**C**) Differential expression patterns by RNA-Seq were confirmed by immunoblotting. ATM, NBS1, CHK1, p53 and p21 antibodies were used for immunoblotting. GAPDH antibody was used as loading control. Immunoblotting data were obtained using C-digit as digital data. Full scan data of each band are provided in Supplementary Figs 3 and 4. RNA-Seq = RNA sequencing, iPSCs = induced pluripotent stem cells, NPCs = neural progenitor cells, HR = homologous recombination, NHEJ = nonhomologous end joining, FPKM = fragments per kilobase of exon per million reads mapped.

DDR activity is accompanied by the maintenance of a high transcriptional level in PSCs [31]. To elucidate the transcriptional differences of DDR genes after reprogramming and differentiation, we performed RNA-Seq analysis using a next-generation sequencer (Illumina HiSeq 2500) with fibroblasts, iPSCs (NB1RGB C2 clone) and NPCs (Fig. 4A and 4B). RNA samples were extracted 1 h after 5 Gy IR exposure. We found that DDR genes, such as those responsible for DNA repair, apoptosis, cell cycle checkpoints and the centrosome were up-regulated in iPSCs (Fig. 4A and 4B, Supplementary Fig. 2). The main DNA repair factors (e.g. *NBS1 [NBN]*, *MRE11 [MRE11A]*, *BRCA1* and *BRCA2*), cell cycle checkpoint factors (e.g. *ATM, ATR, CHK1* and *CHK2*), and apoptosis factors (e.g. p53 [*TP53], CASP3* and *BID*) were up-regulated in iPSCs (Fig. 4B). Interestingly, we observed some patterns in the transcription levels: (i) moderate increases during reprogramming and differentiation in genes such as *NBS1, BRCA1* and *ATM* and (ii) significant up-regulation in reprogramming but down-regulation after differentiation in genes such as *MRE11*, *BRCA2*, *CHEK1*, *CHEK2*, *TP53*, *CASP3* and *BID*. In contrast, the CDKN1A transcription level significantly decreased. The transcriptional product of the *CDKN1A* gene, the p21 protein, is a downstream signaling factor of ATM and p53 in DDR. The expression of these genes was confirmed by western blotting (Fig. 4C and Supplementary Fig. 3). After DNA damage occurs, ATM phosphorylates p53 at serine 15 to activate p21 expression, which inhibits cyclin-dependent protein kinase 2 (CDK2) for G1/S cell cycle checkpoint activation. Despite the high p53 expression, the attenuated p21 expression suggests high apoptosis activity without the G1/S cell cycle checkpoint in iPSCs.

### IR sensitivity and DDR in iPSCs

**Fig. 5:**
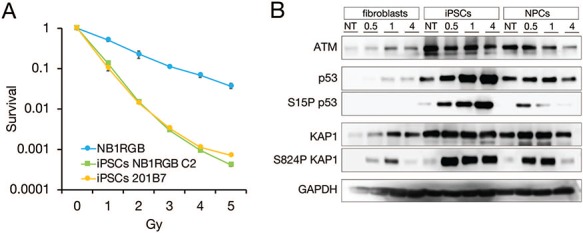
**IR sensitivity and persistent activation of DDR after IR in iPSCs**. (**A**) To determine cell sensitivity to IR exposure, colony formation assay was performed. Fibroblasts (NB1RGB) and iPSCs (NB1RGBC2 and 201B7) were used. Compared with fibroblasts, iPSCs showed hypersensitivity to IR exposure. Error bars represent the SEM. (**B**) After IR exposure, cell extracts were immunoblotted with ATM, p53, serine 15 phospho-p53, KAP1 and serine 824 phospho-KAP1 antibodies. GAPDH antibody was used as a loading control. Immunoblotting data were obtained using C-digit as digital data. Full scan data of each band are provided in Supplementary Fig. 4. DDR hyperactivation increased in a time-dependent manner in iPSCs but not in fibroblasts and NPCs. IR = ionizing radiation, DDR = DNA damage response, iPSCs = induced pluripotent stem cells, SEM = standard error of the mean, ATM = ataxia-telangiectasia-mutated, KAP1 = Kruppel-associated box domain (KRAB)-associated protein-1, GAPDH = glyceraldehyde 3-phosphate dehydrogenase.

To investigate the cellular sensitivity to IR exposure of iPSCs, we performed colony formation assays at designated doses using fibroblasts (NB1RGB) and iPSCs (NB1RGB C2 and 201B7). As expected, iPSCs showed hypersensitivity to IR exposure compared with fibroblasts (Fig. 5A). Furthermore, western blot analysis revealed that persistent p53 phosphorylation at serine 15, which is involved in activation of apoptosis and phosphorylation of KAP1 at serine 824, is associated with chromatin status and DDR in iPSCs but not in fibroblasts and NPCs (Fig. 5B and Supplementary Fig. 4). Both phosphorylations are catalyzed by ATM kinase. GAPDH was used as a loading control. Interestingly, a background level of phosphorylated p53 was observed in iPSCs, suggesting that iPSCs undergo apoptosis to remove endogenously DNA-damaged cells. This observation is consistent with RNA-Seq data and previous reports on constitutive up-regulation of apoptotic genes [29].

## DISCUSSION

We compared DDR and the apoptosis activity between PSCs and differentiated cells. For that purpose, we generated iPSCs from human skin fibroblasts NB1RGB and NPCs from iPSCs as differentiated cells. Human iPSCs 201B7, generated by Takahashi *et al*.,[6] were used as a control cell line. To assess DDR activity, γ-H2AX and 53BP1 antibodies were used for immunostaining. Some researchers have reported that γ-H2AX- and 53BP1-positive foci disappear faster in iPSCs than in differentiated cells 4–8 h after IR exposure, suggesting that iPSCs have a high DDR activity, which allows rapid repair of induced DNA damage [32–34]. However, our data show that γ-H2AX-positive cells remain in iPSCs (52.5% NB1RGB C2 and 54.7% 201B7) and at lower levels in fibroblasts and NPCs (32.3% and 22.3%, respectively) at 4 h after IR. 53BP1-positive cells remain in iPSCs (40.0% NB1RGB C2 and 42.5% 201B7) and at lower levels in fibroblasts and NPCs (22.5% and 14.3%, respectively) at 4 h after IR. These differences might be explained by cell line variations: some groups compared DDR using fibroblast and iPSC cell lines of different lineages, whereas our data show DDR in cell lines from the same lineage. Cell lines from the same lineage share epigenetic backgrounds (for example, chromatin silencing), a factor important for the comparison of DDR. Furthermore, we observed apoptosis-like γ-H2AX foci in iPSCs after IR exposure that increased in a time-dependent manner. We observed the same result with the TUNEL assay in fibroblasts and iPSCs. These results suggest that the apoptosis pathway dominates DNA repair in iPSCs, though both activities are very high. Hence, we speculate that to maintain genome stability, iPSCs with damaged DNA are dominantly removed from the cell population and that cell competition theory might be ongoing in iPSCs.

To address transcriptional regulation in iPSCs and differentiated cells, RNA-Seq was performed. As previously reported, the expression level of DNA repair factors is high in iPSCs and NPCs compared with fibroblasts [31]. Interestingly, although the expression level of some DNA repair factors remains high in iPSCs and NPCs, the expression level of apoptosis-related genes returns to that found in fibroblasts and NPCs. This result suggests that a high apoptosis rate is specific to iPSCs. Consistent with this finding, p53 phosphorylation at serine 15, an apoptosis-activated signal, increased at 4–8 h after IR in iPSCs, whereas it peaked at 0.5 h in NPCs and then decreased. The persistent increase of p53 and KAP1 phosphorylation might suggest a low activity of phosphatase to release DDR. In contrast, p21 expression is very low in iPSCs, suggesting that iPSCs maintain high proliferation to make up for cell loss due to apoptosis caused by endogenous or exogenous genotoxic stress. However, in the absence of the G1/S checkpoint, DNA repair is not facilitated efficiently despite high CHK1 and CHK2 expression. Furthermore, we observed differential expression patterns in the same pathways of DDR during differentiation. MRE11, RAD50 and NBS1 form a complex called the MRN complex, which initiates the HR repair pathway [35–37].

In summary, our study revealed transcriptional changes related to DDR and apoptosis after DNA damage in PSCs. The constitutively activated apoptosis pathway dominantly removes cells with DNA damage to maintain the intact genome DNA of the PSC cell population. In addition, differential expression patterns of NBS1 and MRE11 during differentiation to NPCs might be associated with a different neural disease phenotype of NBS and ATLD. The present findings might help protect genome stability and prevent genomic mutation in regeneration therapy. However, the molecular mechanism underlying transcriptional regulation in DDR needs further investigation.

## Supplementary Material

Supplementary_figure_1_rrz057

Supplementary_figure_2_rrz057

Supplementary_Figure_3_rrz057

Supplementary_Figure_4_rrz057
