# Supplementary figures and images for: Reprogramming and differentiation-dependent transcriptional alteration of DNA damage response and apoptosis genes in human induced pluripotent stem cells
Source: J Radiat Res. 2019 Oct 28;60(6):719–28. doi: 10.1093/jrr/rrz057 (PMC7357234; doi:10.1093/jrr/rrz057)

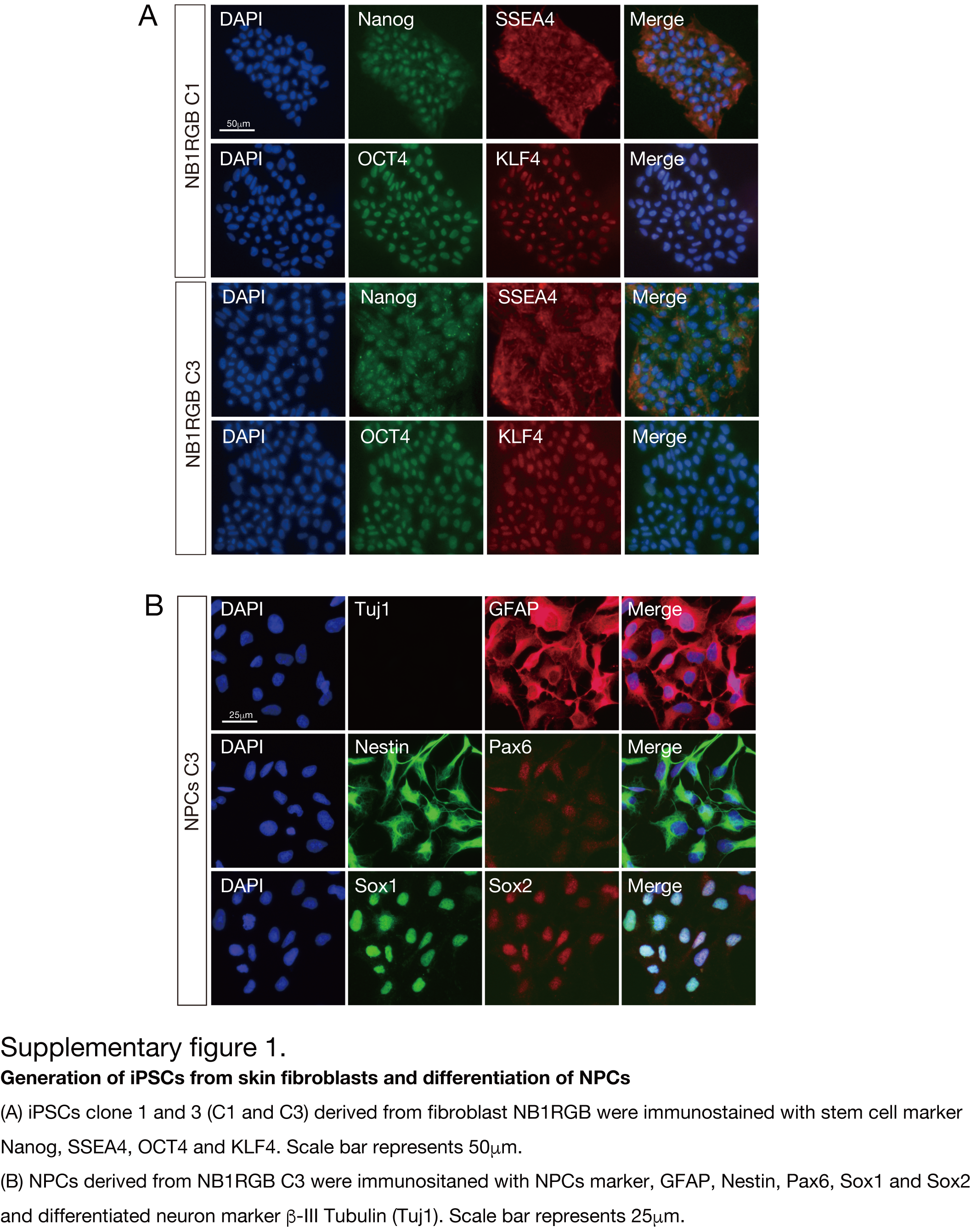

Supplement: Supplementary_figure_1_rrz057 [file supplementary_figure_1_rrz057.png]

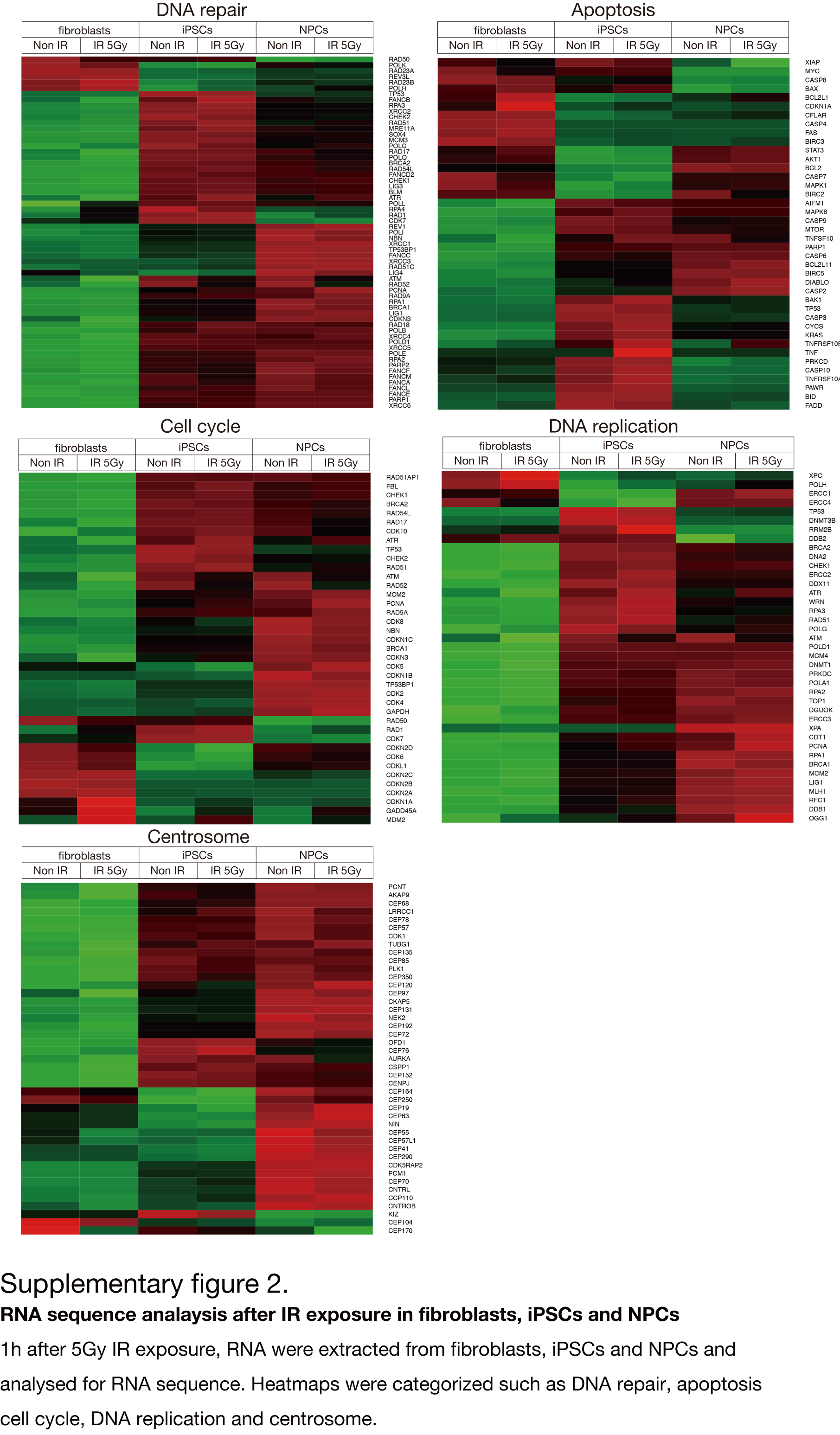

Supplement: Supplementary_figure_2_rrz057 [file supplementary_figure_2_rrz057.png]

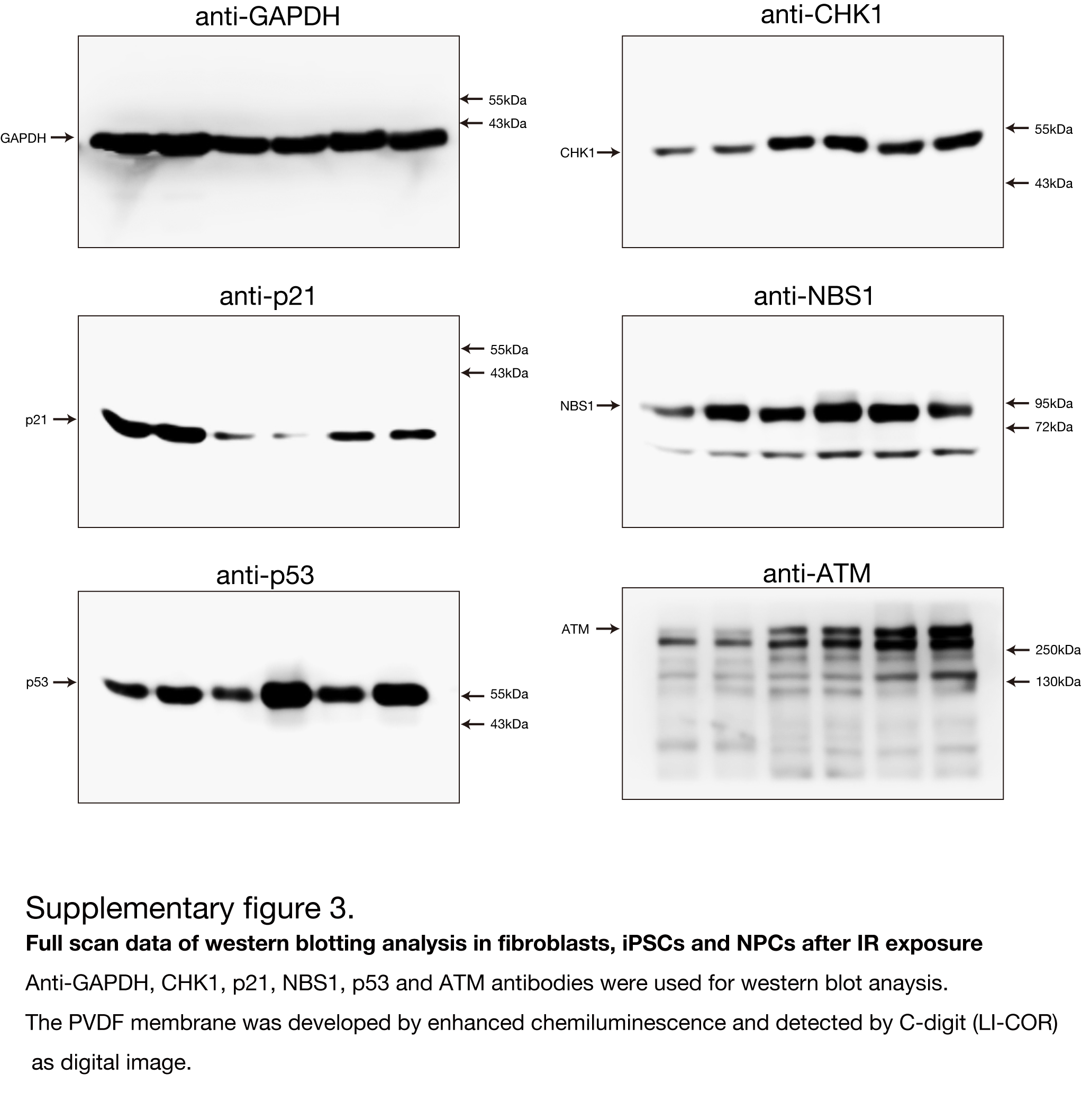

Supplement: Supplementary_Figure_3_rrz057 [file supplementary_figure_3_rrz057.png]

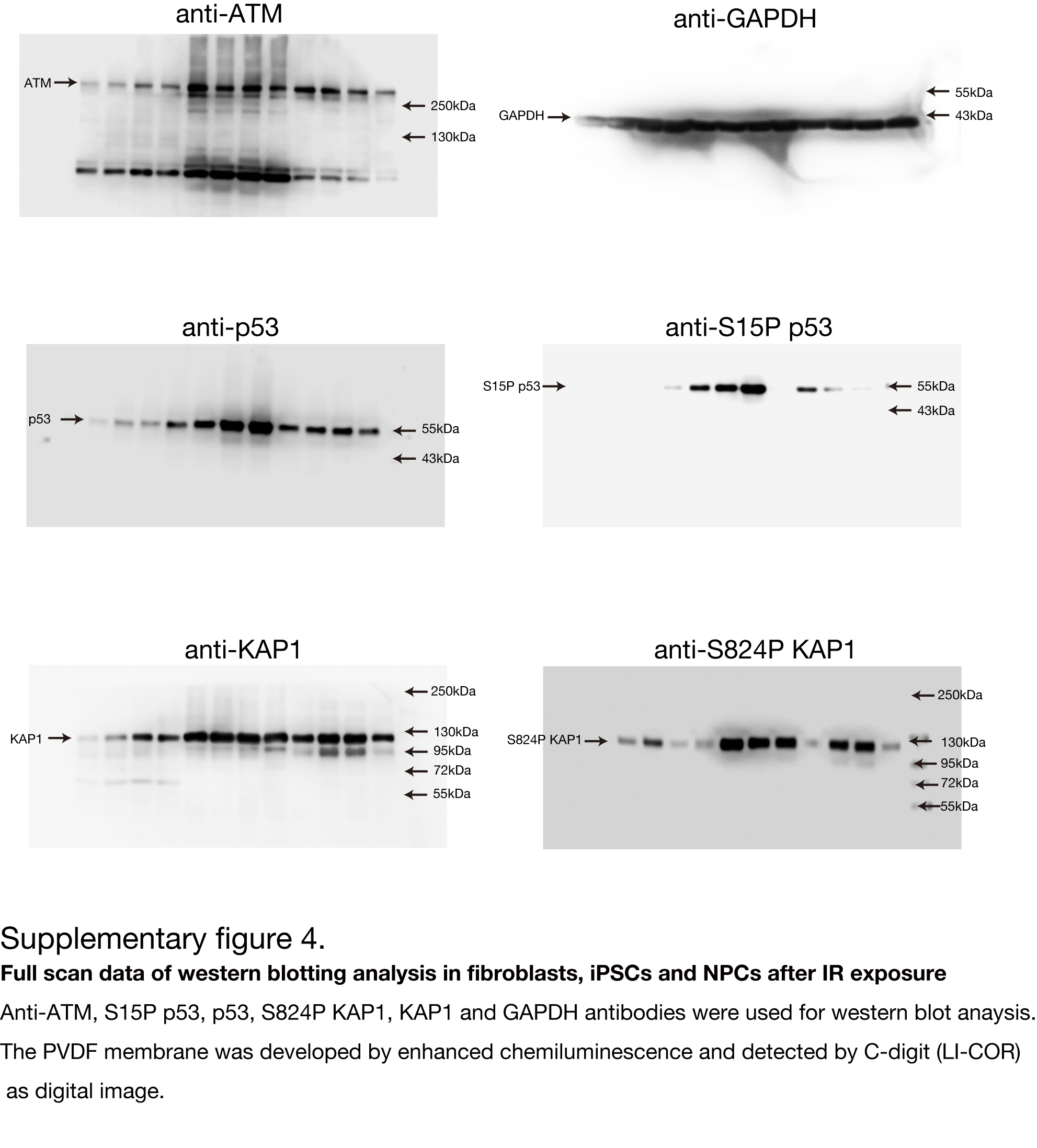

Supplement: Supplementary_Figure_4_rrz057 [file supplementary_figure_4_rrz057.png]
